# The Effects of Meaning-centered Play on Spiritual Sensitivity of Children: A Randomized Controlled Trial

**DOI:** 10.30476/IJCBNM.2021.90120.1669

**Published:** 2022-01

**Authors:** Maryam Fazlollahi, Monir Ramezani, Seyed Mohsen Asghari Nekah, Azadeh Saki, Mohammad Jafar Jahangir Feyzabadi

**Affiliations:** 1 Department of Pediatric Nursing, School of Nursing and Midwifery, Mashhad University of Medical Sciences, Mashhad, Iran; 2 Nursing and Midwifery Care Research Center, Mashhad University of Medical Sciences, Mashhad, Iran; 3 Department of Counseling and Educational Psychology, School of Education and Psychology, Ferdowsi University of Mashhad, Mashhad, Iran; 4 Department of Epidemiology and Biostatistics, School of Health, Mashhad University of Medical Sciences, Mashhad, Iran; 5 Social Determinants of Health Research Centre, Mashhad University of Medical Sciences, Mashhad, Iran; 6 Department of Islamic Instructions, School of Medicine, Mashhad University of Medical Sciences, Mashhad, Iran

**Keywords:** Children, Nursing, Play, Sensitivity, Spirituality

## Abstract

**Background:**

Spiritual sensitivity is the child’s ability to be spiritually aware of what is happening both outside and within him or herself, and the further ability to respond accordingly. On the other hand, play is one of the strategies to develop children’s capabilities. Yet, there is limited information about the relationship of play and spiritual sensitivity. The present study aimed to evaluate the effects of meaning-centered play on children’s spiritual sensitivity.

**Methods:**

This two-group randomized controlled trial was conducted on 120 children aged 10–11 who were recruited from two centers affiliated to the institute for intellectual development in Mashhad, Iran, by convenience sampling between May 2016 to January 2018. Samples were allocated to intervention (60) and control (60) groups through random allocation. In the intervention group, a meaning-centered play intervention was implemented in twelve 45-minute sessions, twice a week and for six weeks. The control group had the usual trend of the center’s plays and programs. Spiritual sensitivity was measured via the Spiritual Sensitivity Scale for Children (SSSC) before and after the end of the intervention. The data were analyzed via the SPSS software (v. 20.0) using Chi-square, independent-sample *t* test, Mann-Whitney U and Wilcoxon signed-rank tests. The significance level was less than 0.05.

**Results:**

Although the groups did not significantly differ from each other concerning the pretest mean scores of spiritual sensitivity and its subscales (P>0.05), the posttest values of these scores in the intervention group were significantly greater than the control group (P<0.05). After the intervention, the mean scores of spiritual sensitivity significantly increased in the intervention group (Before: 65.0±13.6, after: 79.4±12.3, P<0.001), but no significant increase was observed in the control group (Before: 66.7±14.6, after: 67.4±12.3, P=0.604).

**Conclusion:**

According to the results, it can be suggested that meaning-centered play program can be used in play room in schools, child care centers and hospitals to improve the spiritual sensitivity among children.

**Trial Registration Number:**

IRCT2017022232733N

## INTRODUCTION

Spirituality is among the most important needs of all human-beings. It is an ongoing search for meaning and purpose in life. It is also considered as an attempt to foster greater sensitivity to self, others, divine beings, and God or a search for what is necessary to become a human or a complete human. ^1^ Spirituality helps reach high levels of cognitive, moral, and emotional development. It strongly affects attitudes, values, and behaviors as well as the biochemistry and physiology of the body. ^2^

Children have an innate capacity for spiritual experiences and this capacity suggests that spirituality is an essential influence in a child’s healthy development. Children seek out and recognize relationships between self and others, and feel these relationships as an expression of an outward movement from the child’s inner being. These three domains of research invited the quest to understand three essential questions surrounding a child’s spiritual sensing, namely: ‘who am I?’; ‘who am I in relation to other people and the world in which I live?’; and ‘who am I in relation to a state of Being that goes beyond the physicality of this world’? ^3^ The necessity to protect spiritual uniqueness is supported by the ‘signature phenomenon’ of a child’s spiritual sensitivity. Spiritual sensitivity is the child’s ability to be spiritually aware of what is happening both outside and within him or herself, and the further ability to respond accordingly. ^3^

As a significant component in health, spirituality has received an increasing attention during recent years; ^4 , 5^ recently, spirituality in early childhood is increasingly recognized and acknowledged to be as an important aspect of their wellbeing and a more appropriate and relevant starting point for intervention. ^6^ But there are only a few studies about how spirituality/spiritual sensitivity develops in a child. Robert Coles performed one of the handful comprehensive studies into spirituality development among children. He collected the data for his study through interviewing children of different countries and with different religious affiliations and found that despite variations among children respecting their cultural and religious backgrounds, most of them had similar concerns respecting spirituality and their ideals. He attributed children’s spirituality to their desire for knowing and reported that spirituality is linked with different aspects of children’s life, particularly emotional and moral attitudes such as shamefulness and guilt. His study showed that the inner desire for spirituality originates from the curiosity about and the preoccupation with discovering the world and hence, this desire is manifested since early childhood. Finally, Coles concluded that spirituality promotes humanity in children and thus, strategies are needed for its development. ^7^ Another study into spirituality among children was conducted by Hay and Nye on children aged 6–11 years and was presented as the Theory of Relational Consciousness. According to this theory, all children are born with an innate spirituality. Such spirituality, called awareness sensing, is not religion-dependent. They also considered spirituality as a relational consciousness which is a developed capacity for getting aware of relationships with self, others, universe, and a transcendent power. Relational consciousness allows people of different ages to reflect on their spiritual experiences, develop their identity, feel worthy, and understand the meaning and the purpose of life. This theory holds that in order to approve their developing awareness of the world, children seek spiritual experience from their significant others. The three types of sensitivity in this model are awareness-sensing, mystery-sensing, and value-sensing. ^8 , 9^ In line with the Relational Consciousness Theory, Bradford noted that children identify and follow relationships between self and others and perceive such relationships as the external expression of the internal world. Thereby, the community dimension of spirituality was added to the spiritual sensitivities of children. ^10^

Play is one of the strategies to develop children’s capabilities. It has significant roles in developing their social skills and creativity. ^11 - 14^ Moreover, it is a means for improving emotional- behavioral problems. ^15^ Meaning-centered play program was designed to develop the spiritual sensitivity of children through play. The aim of this play program was to familiarize children with spiritual concepts, strengthen their meaningful relationships, foster their awareness, and finally develop their spiritual sensitivity through play. There is limited empirical information, if any, about the relationship of play and spirituality in children. In this case, the results of the study came to the conclusion that the effect of prayer painting on children’s spiritual life requires more studies. ^16^ It should be noted that there are no statistics on children’s spiritual sensitivity due to limited studies. Thus, due to the necessity to design age-appropriate interventions to cultivate the spiritual sensitivity of children; also the importance of pay attention to spirituality in early childhood, the present study was conducted to evaluate the effects of meaning-centered play on children’s spiritual sensitivity. 

## METHODS

This two-group randomized controlled trial was conducted from May 2016 to January 2018. In this study, samples and statistical analysts were blinded. Blinding for the statistical analyst was performed by encoding the data for the intervention and the control group. Therefore, the statistical analyst was unaware of which group the data belonged to. Regarding the samples, considering that the Center for Intellectual Development has play programs, the intervention group and the control group did not know in which play group (meaningful play or center play) they were assigned. It is nothworthy that the intervention group, in addition to the center routine program, received meaning centered plays.

Study setting was two centers affiliated to the Institute for Intellectual Development in Mashhad, Iran. These two centers were selected from the total eight intellectual development centers in Mashhad, Iran, due to their authorities’ closer collaborations. Both centers were similar in terms of services and programs. One of these centers was randomly allocated, through the simple randomization with coin-flipping method, to the intervention group and the other to the control group. Such allocation method was used to prevent between-group information leakage. Then, samples were conveniently recruited from each center. It should be noted that these centers are looking for ways to fill children’s leisure time. Among the activities of these centers are providing education and entertainment, playing, studying, and showing movies and theater performance.

Sample size was calculated based on the findings of a pilot study. Accordingly, with a type I error of 0.05, a type II error of 0.2, a *d* of 4, and a standard deviation of 7.8, the sample size calculation formula determined that sixty samples in each group were needed for the study. 

$N=\frac{{\left(Z_{1-\alpha /2}+Z_{1-\beta }\right)}^{2}2\delta^{2}}{d^{2}}=\frac{{\left(1.96+0.84\right)}^{2}27.8^{2}}{4^{2}}\approx 60$

**Figure 1:**
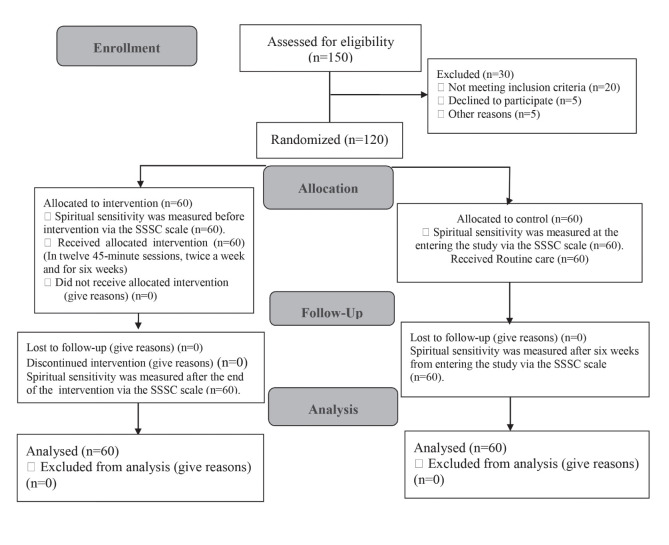
CONSORT Flow Diagram

Convenience sampling was done to recruit eligible children from all children referred to the study setting. Eligibility criteria were children’s age between 10-11 years, no serious physical or mental health problems, living with both parents, having no special educational needs, having tendency to participate in the study, and signing the informed consent by children’s parents. Based on the Spiritual Sensitivity Scale for Children (SSSC) which measures spiritual sensitivity of children aged 8–11 years, the children in the last years of elementary school was chosen. Because these children could understand the meanings of the items of spiritual sensitivity scale and could complete the questionnaire without the intervention of the researcher. This age range, capture the developmental stage of concrete–conceptual thinking. Selected samples were excluded if they developed serious health problems during the study or failed to attend one or more sessions of the study intervention (Figure 1). Samples from the intervention group were matched with their counterparts in the control group respecting their sex. 

The data collection instruments were demoghraphic questionnaire and the SSSC. The demoghraphic questionnaire consisted of 4 items which were: age, birth rank, sex, and parents’ educational level. The SSSC scale was developed by Stoyles et al. based on the studies conducted by Hay and Nye and Bradford, and measures spiritual sensitivity of children aged 8–11 years in the four dimensions of awareness-sensing (6 items), mystery-sensing (5 items), value-sensing (7 items), and community-sensing (5 items). The 23 items of the scale are scored on a four-point scale as follows: 1: “Disagree”; 2: “Slightly agree”; 3: “Agree”: 4: “Completely agree”. Thus, the possible total score of the scale may range from 23 to 92. A higher score indicates greater spiritual sensitivity in child. Face validity of this scale was examined with the sample of nine male and 10 female primary school students ranging in age from 8 to 11 years. The internal consistency of a one-dimensional version of the SSSC was acceptable (Cronbach’s alpha=0.77). Also, Spearman correlations were computed to assess the relationships between spiritual sensitivity, self-esteem and hope (r_s_=0.33, P<0.001 and r_s_=0.56, P<0.001, respectively) (3).

The SSSC scale had not already been translated into Persian and thus, we translated it through the forward-backward translation method. First, two translators independently translated the scale from English into Persian and then, a third translator together with the research team compared translations and generated a single version. The final Persian translation was back-translated into English by two other translators. The two English translations were compared and merged into a single translation by a third translator and the research team. Finally, the similarity between the produced English translation and the original version of the scale was confirmed. After that, ten children from the target population confirmed the face validity of the Persian version of the scale and ten experts in Islamic sciences, pediatric psychology, pediatric nursing, and instrument development confirmed its content validity. Persian version of SSSC showed high content validity ratio of individual items (CVR range: 0.8-1) and high overall content validity index of the SSSC (S-CVI/Ave: 1). Moreover, the reliability of the scale was assessed using the internal consistency assessment method through which fifty children aged 9–11 were recruited from a school in Mashhad, Iran, to complete the scale. Then, Cronbach’s alpha was calculated which was equal to 0.719. Also, the Cronbach’s alpha for the scale dimensions is, respectively o.72, 0.7, 0.75, 0.77.

While children in the control group were treated as all children who referred to the study setting, their counterparts in the intervention group were provided with a meaning-centered play intervention in eight six-person, one five-person, and one seven-person groups (with respect to sex segregation). Questionnaires were distributed and collected by the researcher (first author). The questionnaire was completed by the child. Play sessions were held for each group, according to the planning and coordination with the members of that group. Play sessions were led with the researcher (first author).The intervention for all these groups consisted of twelve 45-minute sessions. Sessions were held twice weekly and the intervention lasted 6 weeks. It should be noted that the intervention group also had routine programs of the center. The aims of the intervention were to familiarize children with spiritual concepts, strengthen their relationships, foster their awareness, and develop their spiritual sensitivity through play. Thus, the intervention was developed based on the four types of spiritual sensitivity, i.e. awareness-sensing, mystery-sensing, value-sensing, and community-sensing (8, 3, 10) and the six criteria for nurturing spirituality as proposed in the Relational Consciousness Theory, i.e. space, process, imagination, relationship, intimacy, and trust. ^8^ The underpinning assumption of the intervention was that spirituality is an existential capacity from the birth with a core of relational consciousness which is related to one’s relationships with self, others, the world, and a transcendent power. ^8^ Accordingly, before the intervention, the SSSC was used to assess spiritual sensitivity in both groups. Then, the meaning-centered play intervention was implemented for children in the intervention group (Table 1 for the detailed information about the intervention).

**Table 1:** The content of the intervention sessions

Session	Concepts	The content of play	Post-play tasks
1–2	Respect; freedom; negotiation; judgment; equity; justice	Communication among participants and facilitators (i.e. the first author and an expert in child education) through happy activities Creative drama: Pictures related to the intended concepts were shown to participants and they were asked to play a show based on them. Whenever needed during play, we provided examples or explanations to make the intended concepts more objective and tangible.	Feelings notebook and personal characteristics: A beautiful notebook, the feelings notebook, was given to each participant at the end of the first session and they were asked to write their feelings about themselves. Besides they were asked to write in a table about their positive and negative physical, moral, and behavioral characteristics and capabilities, interests, dislikes, and self-care activities.
3–4	Thinking; self-knowledge; amazement; collaboration; sympathy; forgiveness	Communication and warm-up: The tables completed in the previous session were collected and participants were asked to think about themselves for minutes. Puppet show: The dolls/pictures of several cartoon characters were given to participants for the purpose of puppet show and comparison with themselves. Then, we aroused their sense of amazement through informing them that each being has its unique characteristics and thereby, directed them towards God. The use of problem-solving, empathy, collaboration, and help techniques: Each participant was required to express one of his/her problems, if any, and the others were required to provide solutions to the problem. Simultaneously, pictures relating to the different types of helping others were shown to participants to familiarize them with helping. Moreover, a story about forgiveness was told to them.	Feelings notebook: Participants were asked to look at their parents, listen to their voices, and talk to them at home and then, write their good feelings about them. In other words, they were asked to write a letter to them. Moreover, they were asked to answer these questions for the next sessions: Who do you like more? Who do you like to like you more?
5–6	Love; beauty; family; friends	Communication and warm-up Imagination seat play: Participants sat on soft inflatable chairs and while listening to sea sound, were asked to imagine that they are at beach with all their beloved ones for a birthday party. They were also asked to imagine that they are looking at the sea and its waves, sensing water touching their feet, and watching the sunset and thereby, they were directed towards God. Handiwork making and storytelling: Then, a story about their families was told to them and they were provided with play dough, paper, and cardboard to make handiworks and tell stories about them.	Feelings notebook: Participants were asked to look at their hands and other body parts, think about them and their mysteries, and say what would happen if they did not exist. Moreover, they were asked to look at their parents eyes and write about their beauties and mysteries. Similarly, they were asked to write their feelings about the beauties of the world, humans, animals, and plants.
7–8	The beauties of the world; the supreme being	Communication and warm-up Looking at flowers and watching movie: Several beautiful flowers were given to participants and they were asked to carefully look at them and their parts and colors, touch them, and express their feelings. Meanwhile, we used directed sentences and words to arouse their senses of amazement and mystery. Then, a movie was shown to them containing the beauties of the world such as sunrise, sunset, sea, stars, plants and animals. The movie also contained a light music and verses from the Holy Quran. Painting: Finally, a large cardboard was given to participants to paint together the most mysterious being in the world.	Feelings notebook: Participants were required to write their feelings about the most supreme power in the world, i.e. God, and write or paint about His favors. Finally, a beautiful flowerpot was presented to each of them.
9–10	Enjoying eating; concentration; thinking about God	Communication and warm-up Concentration: Healthy and tasty foodstuffs were given to participants and they were asked to say the name of their creators, i.e. God, and start eating them calmly while sitting around a table. During eating, they were encouraged to think about the types and the tastes of what they were eating. At the end, they were taught to thank God and were encouraged to consciously do their other daily activities in the same way. Meanwhile using water, they were similarly taught to say the name of God, remember the cleanliness of water, and perform ablution while sensing, touching, and thinking about water and concentrating on their feelings about it. Yoga seat game: Participants were asked to sit on yoga seats, close their eyes, and think about nothing except for God. They were allowed to say the word “God” for the purpose of deeper concentration.	Feelings notebook: Each participant was asked to write a letter to God and also to think about and write his/her most fundamental questions.
11–12	Establishing relationships with self, others, the world, God	Communication and warm-up Imagination seat play: Each participant was asked to sit on an inflatable chair, close his/her eyes, go deep inside his/her body, and imagine himself/herself in close relationship with other elements in the world, and also imagine parents, friends, animals, plants, and the world. After returning from his/her imaginative trip, he/she was asked to express his/her feelings. Group singing: The transcript of a song was given to participants and they were asked to sing it in accordance with music.	Participants read their feelings notebooks for their peers at personal will.

In each group, children (alone) participated. At the beginning of each session, fifteen-minute explanations were provided to samples about the aims of the sessions as well as the rules and strategies to foster intimacy and strengthen relationships among children. Then, the play intervention was implemented and finally, discussions were held on the play and questions were asked from samples about the play in order to explain the different aspects of the intended concept(s). The elements of the games were Persian stories, music and video clips, and painting. The intervention was developed through seeking the comments of several game designers and clinical pediatric psychologists (affiliated to the study setting), spirituality experts, and movie writers. After the study intervention, all samples in the intervention groups were required to re-complete the study instrument. The control group had the current trend of the center’s plays and programs. Spiritual sensitivity of the control group was measured via the SSSC at the beginning of the study and 6 weeks later by first researcher.

This study was approved by the Ethics Committee of Mashhad University of Medical Sciences with the code of IR.MUMS.REC.1395.127. In this study, ethical principles of human research were considered. For the children and their parents, the objectives of the study and how to do the study were explained. Written informed consent was obtained from the parents of the children participating in the study. It was explained to the children and their parents that they could withdraw from the study whenever they wished. The names of children were kept confidential. All information was analyzed and reported anonymously and coded.

The data were analyzed via the SPSS software version 20.0. First, the Kolmogorov-Smirnov test was run to test the normality of the study variables. Then, the paired t-test (for data with normal distribution) and Wilcoxon signed-rank test (for data without normal distribution) were conducted for within group comparisons; also,independent-sample t-test(for data with normal distribution) and Mann-Whitney U (for data without normal distribution) were conducted for between-group comparisons. Chi-square test was applied for qualitative variables. All statistical tests were performed at a significance level of 0.05.

## RESULTS

All 120 recruited participants completed the study. Most participants in both groups were female. The mean age of the participants in the intervention and the control groups was 10.4±0.5 and 10.6±0.5, respectively. There were no statistically significant differences between the study groups in terms of age (P=0.71), sex (P=0.5), birth rank (P=0.69), and their parents’ educational levels (P=0.06 & P=0.21; Table 2).

**Table 2:** Comparison of the groups concerning the participants’ demographic characteristics

Groups		Intervention	Control	P value*
		N (%)	N (%)	
Characteristics				
Birth rank	First child	41 (68.3)	39 (65.0)	0.69
	Second child	19 (31.7)	21 (35.0)	
Father’s education	Diploma	20 (33.3)	30 (50.0)	0.06
	University	40 (66.7)	30 (50.0)	
Mother’s education	Diploma	13 (21.7)	19 (31.7)	0.21
	University	47 (78.3)	41 (68.3)	
Sex	Male	11 (18.3)	14 (23.3)	0.50
	Female	49 (81.7)	46 (76.7)	

* Chi-square test

The results of the independent-sample *t* and Mann-Whitney U tests illustrated that at pretest, the groups did not significantly differ from each other in terms of the mean scores of spiritual sensitivity and its subscales (P>0.05) (Table 3). The paired-sample t and Wilcoxon signed-rank tests showed that after the study, the mean scores of spiritual sensitivity and all its four subscales significantly increased in the intervention group (P<0.001), while none of them significantly changed in the control group (P>0.05) (Table 3). Consequently, after the intervention, the mean scores of spiritual sensitivity and all its four subscales in the intervention group were significantly greater than the control group (P≤0.001) (Table 3).

**Table 3:** Comparison of the groups concerning the mean scores of spiritual sensitivity and its subscales

Spiritual sensitivity’s subscales		Group		P value
		Intervention Mean±SD	Control Mean±SD	
	Time
Awareness-sensing	Before	15.70±3.6	16.68±4.2	0.37***
	After	20.37±36	16.98±4.2	<0.001***
	P value*	P<0.001	P=0.32	
Mystery-sensing	Before	14.36±3.5	14.38±3.0	0.59****
	After	17.17±3.5	14.8±3.5	<0.001****
	P value**	P<0.001	P=0.24	
Value-sensing	Before	20.35±4.9	20.92±4.9	0.64****
	After	24.45±3.5	21.7±4.2	>0.001****
	P value**	P<0.001	P=0.61	
Community-sensing	Before	14.65±3.5	15.43±3.5	0.23***
	After	17.43±3.0	15.30±3.5	0.001***
	P value*	P<0.001	P=0.21	
Spiritual sensitivity total	Before	65.0±13.6	66.7±14.6	0.51****
	After	79.4±12.3	67.4±12.3	<0.001****
	P value**	P<0.001	P=0.60	

* Wilcoxon signed-rank test;

** Paired-sample *t* test;

*** Mann-Whitney U test;

**** Independent-sample *t* test

## DISCUSSION

This study assessed the effects of meaning-centered play on children’s spiritual sensitivity. Findings revealed that the meaning-centered play intervention significantly improved the mean scores of spiritual sensitivity and all its subscales in the intervention group, while no significant increase was observed in the control group. 

Since holistic care requires attention to all the dimensions of human life, spiritual care interventions should also be among the nurses’ priorities. ^17^ Despite the importance of spirituality in children’s health and well-being, very few studies have been conducted on spiritual care in children, as opposed to adults. ^16^ In previous studies, we could not find any interventional study on the spiritual sensitivity among children for the purpose of comparison. However, there were some studies which were in the same line with ours in some ways. For instance, some interventional studies were conducted on concepts related to spirituality in children. In line with our findings, a study evaluated the effects of story on affection, spirituality, power, behavior, mind, and cognition; it was reported that it could be a good intervention for nurturing children’s spirituality. ^18^ Another study concluded that life skills training can positively affect spiritual intelligence among female high-school students. ^19^ Similarly, a study on homeless young people in shelters in the United States found that their participation in a meditation-based Youth Education in Spiritual Self-Schema program significantly improved their spiritual health. ^20^ Like our study, all these studies used meaning-centered interventions and reported their effectiveness in promoting spirituality. In line with our findings, another study noted that the use of philosophy can help promote the children’s spiritual intelligence; of course, the mentioned study provided no practical approach for this purpose.7 However, in our play intervention we included philosophical concepts such as ethics, values, and so on. In fact, we used the child’s mind to learn and pay attention to moral and value concepts through play. Contrary to the present study, the result of one study showed that 6 sessions of prayer painting in 6 consecutive days had no significant effect on children’s spiritual life and it requires more studies to be conducted in this field. ^16^ In justifying this discrepancy, it can be pointed out that spirituality is a complex and multidimensional concept in children and it seems that a one-dimensional intervention (prayer painting) cannot lead to a significant change in children’s spirituality in short time.

Regarding the subscales of spiritual sensitivity in children, we found that the meaning-centered play intervention significantly improved the mean score of the mystery-sensing subscale of spiritual sensitivity. This finding may be due to the effectiveness of the intervention in strengthening the participants’ relationships with self, nature, world, others, and God. Similarly, an earlier study reported the positive long-term effects of poem-based make-believe play on creativity among parentless girls, denoting that creativity can be promoted through imagination, play, and poem. ^13^ One of the components of our intervention was creative drama. In the past years, by emphasizing the humanization of the educational process, lots of efforts for reformation of the traditional education system for children were seen in connection with changes in society. These efforts are in line with the requirements of the present time. The educational process needs to be organized in a way which ensures individual development of a person to a full extent of his or her capabilities in order to prepare him or her for the best possible integration into society. In this regard, creative drama represents and emphasizes the individuality of every person and shapes life. It is based on learning through experience, uses experiential and cooperative teaching during creative activities which enhance the development of child’s personality, and has a creative approach to the surrounding world. ^21^ A former study found that the placement of children in creative drama situations significantly improved their imagination. ^22^

Another finding of the present study was the effectiveness of the study intervention in significantly improving the children’s value-sensing ability. A former study also reported the importance of child-led engagement in play as underpinning values. ^23^ Given the potential developmental significance of play, it is important to provide high-quality opportunities for children to engage in play. One of the features of outdoor playtime which mostly supports independent development is the relative lack of adult supervision compared to that found in classroom contexts. ^23^ Therefore, interventions designed to give meaning and identity to child play and capitalize on the inherent developmental opportunities of playtime may be most effective when they can preserve the child-led focus, rather than, say, introducing adult-led sports activities. Child-led engagement in play aims at the promotion of thinking and reasoning. Similarly, the aim of our meaning-centered play intervention was to encourage the children to think about different spiritual issues such as values in play activities. 

The other finding of the study was improvements in the participants’ community-sensing ability following the implementation of the meaning-centered play intervention. Playtime has been associated with the opportunity to develop friendships, which is in turn related to children’s sense of social identity and well-being. ^23^ In agreement with this finding, a former study reported that play had a significant relationship with the development of children’s social skills. ^11^

The findings of this study showed that the participants’ awareness sensing was improved after the intervention. In the case of awareness-sensing, there are approaches which a person can choose to pay attention his/her affairs, such as “attending to the present moment”, and “getting in touch with the felt sense of reality”. In this way, it is notheworthy that in Catholic schools, the raising of awareness of the spirituality is a significant component of the curriculum. ^24^ Also, the need to allocate time in the school day to personal reflection and re-orientation is seen by the trend in teaching young people’s ‘mindfulness’ practices. Such practices may be helpful for the search for a sense of meaning. ^25^

Accurate design of games, participation of various inter-professional experts, and the use of standard and age-appropriate tool to measure the children’s spiritual sensitivity are the strengths of the present study. There was a possibility that the cultural backgrounds of the children’s families were different. Precise control of this case was beyond the power of the researcher. Also, self-reporting of the study tool can be one of the limitations of the present study.

## CONCLUSION

This study indicated that 12 sessions of meaning-centered play in 6 weeks could significantly improve the children’s spiritual sensitivity. Given that Iran has a predominantly religious culture and, as such, spiritual care interventions such as meaning-centered play program are commonplace and consistent with Iranian culture. Thus, this age-appropriate intervention which is compatible with culture can be safely used by care providers and pediatric nurses in kindergartens, schools, wellbeing centers, and play rooms in hospitals in order to improve spiritual sensitivity among healthy and sick children. Also, according to the potentials of this play program, it is suggested that studies should be conducted on the effect of meaning centered play program on children’s creativity, happiness, hope, and quality of life.

## ACKNOWLEDGEMENT

This study was part of a Master’s thesis in pediatric nursing approved by the Ethics Committee of Mashhad University of Medical Sciences, Mashhad, Iran (with the code of IR.MUMS.REC.1395.12). The Authors would like to thank Research Deputy of the University for providing financial support of the study.We are also grateful to the authorities of the Institute for Intellectual Development in Mashhad, Iran, and all children who participated in the study. Research Administration of the Mashhad University of Medical Sciences, Mashhad, Iran (with the code of grant 941659).

**Conflict of Interest:** None is declared. 
